# Do Monetary Incentives Influence Users’ Behavior in Participatory Sensing?

**DOI:** 10.3390/s18051426

**Published:** 2018-05-04

**Authors:** Ngo Manh Khoi, Sven Casteleyn, M. Mehdi Moradi, Edzer Pebesma

**Affiliations:** 1 GEOTEC, Institute of New Imaging Technologies (INIT), Universitat Jaume I, 12071 Castellón, Spain; sven.casteleyn@uji.es (S.C.); moradi@uji.es (M.M.M.); 2 Institute for Geoinformatics, Universität Münster, 48149 Münster, Germany; edzer.pebesma@uni-muenster.de

**Keywords:** participatory sensing, monetary incentive, user behavior, user engagement, smart city

## Abstract

Participatory sensing combines the powerful sensing capabilities of current mobile devices with the mobility and intelligence of human beings, and as such has to potential to collect various types of information at a high spatial and temporal resolution. Success, however, entirely relies on the willingness and motivation of the users to carry out sensing tasks, and thus it is essential to incentivize the users’ active participation. In this article, we first present an open, generic participatory sensing framework (Citizense) which aims to make participatory sensing more accessible, flexible and transparent. Within the context of this framework we adopt three monetary incentive mechanisms which prioritize the fairness for the users while maintaining their simplicity and portability: fixed micro-payment, variable micro-payment and lottery. This incentive-enabled framework is then deployed on a large scale, real-world case study, where 230 participants were exposed to 44 different sensing campaigns. By randomly distributing incentive mechanisms among participants and a subset of campaigns, we study the behaviors of the overall population as well as the behaviors of different subgroups divided by demographic information with respect to the various incentive mechanisms. As a result of our study, we can conclude that (1) in general, monetary incentives work to improve participation rate; (2) for the overall population, a general descending order in terms of effectiveness of the incentive mechanisms can be established: fixed micro-payment first, then lottery-style payout and finally variable micro-payment. These two conclusions hold for all the demographic subgroups, even though different different internal distances between the incentive mechanisms are observed for different subgroups. Finally, a negative correlation between age and participation rate was found: older participants contribute less compared to their younger peers.

## 1. Introduction

An ever increasing number of Internet-connected mobile devices incorporating embedded sensors with powerful sensing capabilities has given birth to the mobile crowdsensing approach [1], which combines the mobility of people and the sensing capabilities of the device. Two variants of mobile crowd sensing are generally discerned: opportunistic sensing, in which the explicit participation of the device owner is not required [2,3,4], and participatory sensing, which is the focus of this article, in which participants explicitly contribute sensing data [5,6]. Due to the proliferation of mobile devices and the active involvement of the participants, participatory sensing applications are able to collect information in a variety of fields [7,8] with high spatial and temporal resolution. For instance, in Smart Cities, we can find participatory sensing applications that measure noise [9,10,11], noise with subjective feedback [12], air pollution [13,14,15], road and traffic condition [16,17], structural health monitoring [18] and cellular signal strength [19]. Recently, some attempts were made to create participatory sensing applications that can flexibly collect different types of data; examples include Ohmage [20], OpenDataKit [21] and Sensr [22].

One of the unique features of participatory sensing is that it entirely relies on participants, who are expected to properly and truthfully collect and submit information to the campaign author in a timely manner. On one hand, it is essential to precisely understand the context in which participants collect the data [23,24,25,26]. On the other hand, participants may be indifferent or even reluctant to contribute this much-needed information due to various reasons, such as the concern of privacy, the cost of mobile data and the extra battery consumption. Therefore, apart from designing a technically good and usable participatory sensing platform, it is also essential to recruit willing and relevant participants [27,28] and subsequently motivate them to actively participate in the data collection. To this aim, incentives, usually in the form of monetary or non-monetary rewards, are considered a key factor in the successful deployment of a participatory sensing application [29]. Such rewards may be based on different data acquisition, user participation, quality or social factors. For example, the distance between the actual location of the submission and the pre-defined point of interest [30], the quality of the submitted data [31], the submission’s contribution to the social welfare [32] and the number of submissions or the efforts spent to produce a submission [33].

Despite the fact that there is an abundance of works on incentives in participatory sensing, most of them consider the topic from a theoretical perspective [34,35,36,37], and present an incentive mechanism, e.g., by mathematical models [31] or computer simulation [30,38]. As acknowledged by a recent survey [37], there is a clear need for empirical studies on the interaction between participants and incentive mechanisms of participatory sensing applications in a real-world context. To the best of our knowledge, there are only a few works that actually recorded the results of deploying incentive mechanisms in real-life conditions [39,40,41]. However, these deployments only offered a little insight on how participants perceive and react to the given incentives due to the small number of participants (18 participants in [41] and 36 participants in [40]) and the simple settings of the deployments (only one task throughout the deployment as in [39]).

In this article, we present Citizense, a multi-purpose incentive-enabled participatory sensing framework that allows campaign authors to design and launch participatory sensing campaigns, and participants to view and respond to these campaigns. In order to study the effectiveness of different incentive mechanisms, Citizense was deployed in a large-scale real-world setting, where 230 participants were exposed to a variety of participatory sensing tasks and three different incentive mechanisms (lottery-style, fixed micro-payment, variable micro-payment) over a 20-day time period. We discuss the setup of the experiment, and subsequently present and statistically analyze the results of this large empirical study. As a result, we can conclude that monetary incentives effectively boosted the participation of the users (compared to campaigns without incentive). Furthermore, we proved that the participants preferred fixed micro-payment over variable micro-payment. A smaller scale qualitative analysis also showed that 62.5% of respondents indicated that, from all incentive mechanisms, they prefer fixed micro-payment. In absolute numbers, the lottery style incentive mechanism is ranked in between fixed and variable micro-payment, but no statistically significant difference could be shown.

The rest of the paper is organized as follows: Section 2 reviews and categorizes the existing incentive mechanisms. In Section 3, our Citizense framework is introduced together with the three incentive mechanisms. Section 4 details the setup of the experiment. The statistical analysis of the results is presented in Section 5. Section 6 further discusses the findings from the statistical analysis. Finally, Section 7 presents the final conclusions.

## 2. Related Works

### 2.1. Classification of Incentives

The participatory sensing process is often hampered by natural factors such as battery life, more occupied memory in the participants’ mobile devices and more mobile data consumption. Furthermore, the participants are adversely affected by their privacy concern as this participatory sensing process might reveal their identity, location and mobility patterns; they might be traced by the collected sensor readings [42]. As a result, incentives are believed to motivate participants by directly compensating them for their various costs resulting from the participatory sensing process and at the same time maintain the trustworthiness of the results [43]. In some special cases, apart from the reward for his/her own submission, a participant can be indirectly awarded based on the performance of other peers; the latter are recruited into the participatory sensing scheme by the recommendation of the former [44]. This policy is reported to simultaneously improve the welfare of several participants and increase the amount of participation.

Due to their potential, there is an abundance of works on incentives in participatory sensing. At the same time, there are some attempts to categorize these incentives based on their different dimensions [34,35,36,37]. In [37], the incentives are classified into application-specific incentives or general-purpose incentives based on their characteristics. Application-specific incentives are specialized mechanisms adapted to a specific participatory sensing application; they are not designed to be generic and thus cannot be applied in other applications. An example for this category is LiveCompare [45], an application that utilizes the quid-pro-quo interactions among its users; they exchange photos of their common product of interest. In contrast, general purpose incentives, such as those studied in this article, can be applied to a wide range of participatory sensing applications. This category of incentives, which are much more common among participatory sensing applications, are further categorized into non-game theoretical and game theoretical incentives. This division is based on the use of mathematical models that represent the rational decision making process. For example, compete micro-payment as in [39] represents the non-game theoretical incentive category. Inside the game-theoretical category, there is a distinction between auction-based and non-auction-based incentives. For auction-based incentive, the examples are reverse auction-based RADP-VPC [46] and TSCM [47] while an example of non-auction-based incentive is the reward-based collaboration scheme [48].

In the work of Jaimes et al. [35], the incentive mechanisms are categorized based on the type of incentives being used; the two main categories are monetary and non-monetary incentives. In the category of monetary incentives, there are two sub-categories of static monetary incentives and dynamic monetary incentives. Examples are macro-payment [39] and uniform payment [40] for static monetary incentives and RADP-VPC [46] for dynamic monetary incentives. In the category of non-monetary incentives, the division is again based on the type of incentive being used; the three sub-categories are collective motives, social rewards and intrinsic motives and fun. Examples for these three sub-categories are [45,49,50], respectively.

Zhang et al. [36], proposed three incentive categories: entertainment, service and money. With respect to Jaimes et al.’s categorization [35], the first two categories can be branded as non-monetary incentives, while the latter are monetary incentives. Although the authors did not further categorize the incentives in their money category, these incentive can be approximately grouped into three groups: posted price, auction-based and Stackelberg game-based, with examples of [31,39,48], respectively.

Gao and colleagues [34], offered two different dimensions of incentives. They can be categorized by their employment purposes or by their incentive negotiation process. The employment purpose divides the incentives into user-centric and platform-centric groups; the former focuses on improving the recruitment process and the participants’ motivation (e.g., [46]) while the latter focuses on improving the information gain of the platform and optimizing (minimizing) the payments to the participants (e.g., [30]). The incentive negotiation process divides the incentives into “Price-Decision-First” and “Data-Upload-First” groups; the first group allows participants to decide on their rewards before whether or not performing the actual sensing task (e.g., [31,38]) while in the second group participants have to upload the sensing results before any decisions on rewards are made by the platform (e.g., [51]).

In our Citizense framework, we adopted and empirically evaluated three monetary incentive mechanisms (i.e., lottery-style, fixed micro-payment, variable micro-payment) as the main tool to effectively engage the participants. We complemented them with campaigns without incentives, serving as a control group. Furthermore, to maintain the quality of the submissions, these monetary incentive mechanisms require the participants to upload their data first in order to verify the information quality before assigning a reward to the participants.

### 2.2. Deployments of Monetary Incentives in a Real Context

From our literature overview, most of the works we found on incentives in participatory sensing are on the theoretical side. For example, in a very recent survey of incentives [37] that addressed 43 works on the use of incentive, only 4 works discuss the results of real-world deployments of different incentive types. The rest of the works usually rely on computer simulation to propose an incentive model and/or to prove the effectiveness of such incentive model. In these work, the computer simulation depends on several parameters and probability values which are preset or varied within a preset range. For example, in [52], the parameters of the computer simulation are the following: number of device—400 to 1000, number of sensing task—40, cost of each task—0 to 50, maximum number of bids per user—3. In some other works, a mathematical proof is used to validate an incentive mechanism [31]. However, we argue that these methods do not fully simulate the complexity of the participants’ psychological behaviors and other environmental factors that may affect the participants and their interaction with the incentive mechanisms, such as the urgency of the task, the relevance of the task, personal preference or the effect of the “word of mouth”. Therefore, in this article, we focus on the deployments of incentive mechanisms in real life contexts. The main factors that determine the realities of the contexts in which an incentive mechanism is tested are, besides the incentive mechanisms themselves (i.e., type of incentive, the way of awarding, amount of reward), the content the sensing tasks, the diverse background of the participants and the large size of the participant pool.

Through our literature review, we found a couple of deployments of incentive mechanisms targeting human participants [11,39,40,41,53,54,55,56]. A closer look into each deployment reveals its characteristics as well as its shortcomings. In [11], although 49 participants are exposed to a realistic task of measuring the noise level, there is only one task to be completed. Furthermore, virtual credits within the prototype were used to motivate the participants instead of real monetary items. Similar as [11], the research in [39] exposed 55 participants to only one task of taking photos of the trash bin, and each of them have access to only one incentive mechanism among the five mechanisms: lump sum, low micro-payment, medium-micro-payment, high micro-payment and compete micro-payment. Although cash was used as the reward for participants, the relationship between the different content of the sensing task and the incentive mechanism was not studied in this work due to the availability of only one sensing task. In [40], 36 participants were divided into three groups, where each group has a different incentive mechanism assigned that allocates virtual credit instead (rather than real money). The same task of carrying wearable sensors and answering 20 questionnaires per day was performed by each group. In [56] the authors divided the participants into two groups, each was faced with a different incentive mechanism. We argue that the participants in these researches did not have knowledge of other incentive mechanisms apart from the one to which they are exposed, therefore it might be not enough to draw conclusion on the effectiveness of a particular incentive mechanism, particularly in competition with others. Furthermore, compared with [39], the number of participants are low and the diversity of incentive are less. In [41], only 18 participants are involved in a research of combining monetary incentive and gamification-based techniques with the sensing task of gathering information at a specific point of interest. The largest number of participants was recorded in [53] where thousands of participants were involved in a research with extrinsic and intrinsic motivations for crowdsourcing in an enterprise context. Other examples, more specifically in participatory sensing, of works performed in a specific context are [54], where 96 participants of a conference were given a specific task to perform in order to win rewards in the form of micro-payment and a lottery, and [55], where participants were subjected to different incentive mechanisms for producing annotations of astronomical objects. In the last three examples, the participants are a non-heterogeneous group (i.e., employees of the company; conference goers; astronomers) asked to do a very specific sensing task in a particular context; they do not represent the diverse nature of people in a society performing a broad range of sensing tasks.

Our review process has shown that the recent deployments in real-life context of incentive mechanisms mentioned above have suffered from one or some of the following shortcomings: the sensing task does not address a realistic topic or is too specific, the context in which the experiment is too specific, a low number of tasks, a low number of participants and the participants’ exposure to only one incentive mechanism. Furthermore, only two [40,55] of the aforementioned related works substantiate their conclusions with a thorough statistical analysis of the results. Other works [11,39,41,53,54,56] highlight the effectiveness of a particular incentive mechanism by presenting and discussing the results with numbers, percentage and charts, yet without underlying statistical analysis to strengthen conclusions. In contrast, this article presents a case study involving a large number of participant (230) in a real-world context, where the participants are simultaneously exposed to a variety of incentive mechanisms and a variety of realistic tasks (campaigns). Furthermore, we provide a state-of-the-art statistical analysis to substantiate our conclusions.

## 3. The Citizense Framework

For the sake of self-containment and full understandability of the remainder of this article, we here provide a brief, high-level overview of the Citizense framework. Further (technical) details about the design and implementation of the Citizense framework can be found in [57].

**Figure 1:**
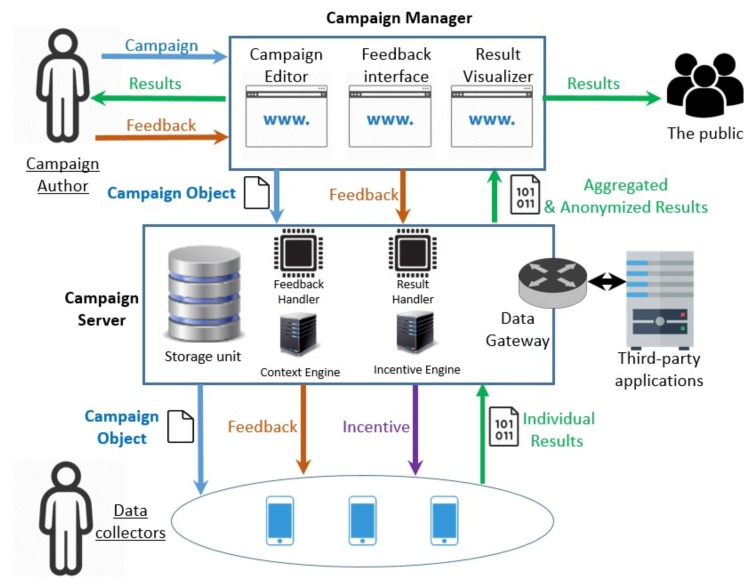
The architecture of the Citizense framework.

Citizense is a flexible and generic user-oriented participatory sensing framework, aiming to ease the process of creating, launching and managing participatory sensing campaigns. The main design concept of Citizense is to decompose a complex domain-specific hardware-dependent sensing process into a list of simple atomic sensing tasks, each task collecting a single type of information or involving a single sensor. As a result, the framework is reusable in different domains and usable by various non-expert users. To realize this concept, the Citizense framework consists of four main components (Figure 1): campaigns, the campaign manager, the campaign server and the mobile client application (app). The two main actors of the framework are the campaign authors (create, launch and subsequently manage the sensing campaign) and the data collectors (execute the campaign by collecting the required data through their mobile devices). Currently the Citizense framework supports 13 different types of atomic sensing tasks, grouped into three groups: sensory input (GPS location, WiFi signal strength and sound measurement), multimedia input (picture) and human input (text input, numeric input, multiple-choice questions, date input and time input).

### 3.1. Campaigns

A campaign (object) is the blueprint of a sensing process, specifying all the necessary parameters for the Citizense framework to be able to visualize, execute and obtain (user-provided) data through the mobile app, communicate campaign and campaign data between server and mobile client app, and store and visualize obtained campaign data on the campaign server.

### 3.2. The Campaign Manager

Serving as the main interface for the campaign authors, the campaign manager allows them to visually and intuitively build a sensing campaign by specifying all the campaign’s necessary parameters such as campaign title, location and time constraints, list of atomic sensing tasks and the (dynamic) transition among them (e.g., fixed transitions, conditional transitions and loops). In order to maintain the quality of information, two measures are currently incorporated in Citizense: (1) the Citizense framework allows the campaign authors to define certain atomic sensing tasks as compulsory to complete (cannot be skipped by data collectors). These compulsory tasks consist of two types: conjunctive and disjunctive; (2) campaign authors may indicate, unknowingly to the data collector, certain atomic sensing tasks as required to receive an incentive. The data collector is not aware of this and these tasks can be skipped, yet doing so will exclude the data collector from receiving an incentive reward for the associated sensing campaign. These measures aim to ensure that essential data is collected on one hand, and discourages users to skip through the atomic sensing tasks to obtain a incentive reward on the other hand. Both compulsory and incentive-required tasks are specified using the campaign manager. Along with creating (and modifying) campaigns, the campaign manager visualizes in real-time the individual and aggregated results from the data collectors. Results are grouped by sensing task and per submission, allowing campaign authors (and the public, if permissible) to understand the results of a campaign through different dimensions. Furthermore, the campaign manager allows campaign authors to send individual or group notifications to data collectors, which are received as notifications on their mobile device. This communication channel can be used to send feedback on submission results, notification of availability of new campaigns, etc.

### 3.3. The Campaign Server

The campaign server is assigned with two main tasks: (1) serving data collectors with context-relevant campaigns; (2) processing the submissions. In the former task, the server matches the context information of the data collectors with those of the available campaigns, then matching campaigns will be sent to the data collectors for execution. In the latter task, the server will process valid submissions based on criteria defined in its corresponding campaign so that the data collector behind that submission will earn experience points and rewards (if applicable).

### 3.4. The Mobile Client App

**Figure 2:**
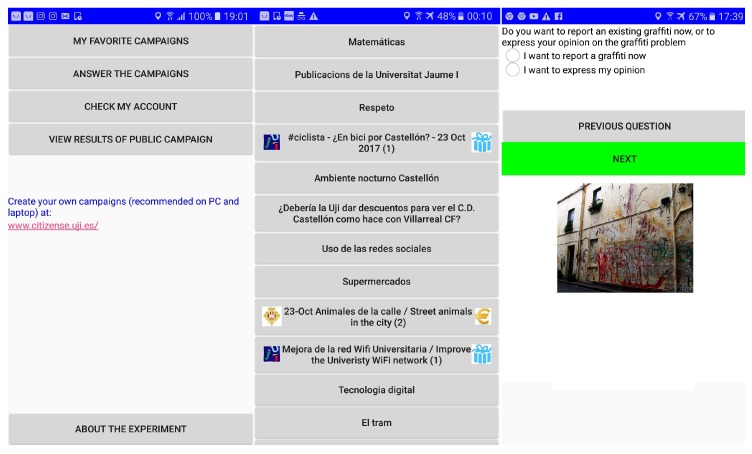
The mobile client application. From left to right: (**left**) the home screen; (**middle**) the list of available campaigns, indicating title, logo of author and incentive, if applicable; (**right**) an atomic sensing task.

Serving as the main interface for the data collectors (Figure 2), the mobile app (currently implemented as an Android app) sends a request and subsequently receives the context-relevant campaigns from the central server, and renders them as a list on the device’s screen (Figure 2 Middle). The list informs the data collectors of the basic information of campaigns: name, logo of the campaign author (if available) and type of incentive. In the next step, the details of the various parameters of the campaign are shown; if the campaign is applied with incentive(s), all the related details of these incentives will be shown. More on visualization and types of incentives can be found in Section 3.5. During the campaign’s execution, the mobile app provides data collectors with suitable controls to produce results for each sensing task (Figure 2 Right). Throughout the process, data collectors can drop out at any moment, i.e., before or during the campaign. At any times, data collectors can query the results of performed campaigns (if permissible), their earned rewards and the author’s feedback using the mobile app.

### 3.5. The Incentive Mechanisms Used in the Citizense Framework

The Citizense framework, among other participatory sensing platforms, is intended to be used in the Smart city context [58], where the participation of all citizens and their sensed information are the ultimate outcomes of our framework. However, throughout our literature review, we observed that a large number of incentive mechanisms focus on optimizing and/or minimizing the amount paid by the participatory sensing platform (or the campaign author). In other words, these incentive mechanisms operate on a market-oriented principle: the participatory sensing platform (or the campaign author) tries to buy sensed information at the lowest possible and/or optimal price, often neglecting other factors such as the fairness among participants or the wellbeing of the participants. In contrast, a “Smart city” encourages the full and equal participation of the citizens [59]. Because of that, in our Citizense framework, we adopt fairness as a main principle when allocating the rewards for the participants: the payment depends on criteria that are theoretically fair to all participants. This principle also avoids the use of auction mechanisms, which are employed by many incentive mechanisms, and introduces additional complexity and communication overhead to the data collectors’ devices [34].

Specifically, the Citizense framework adopts monetary incentives, as this type of incentive has larger applicability in participatory sensing; they are general in purpose and are not bound to any particular participatory sensing scenario and/or application. We use the following monetary incentive mechanisms in the Citizense framework:

- Mechanism $M_{1}$—Lottery: The participants who submitted at least one valid answer to the campaign have the chance to win the reward(s) defined in advance by the campaign author. The campaign server randomly selects the winning submission(s) and assigns the prize to the corresponding participant(s). This mechanism shares some similarities with all-pay auction [60] as they both ask the data collectors to complete the campaign first before the reward is allocated. The difference lies in the selection of the data collector(s); all-pay auction selects the winning data collector(s) based on their performances while mechanism $M_{1}$ randomly selects the data collector(s). This mechanism allows the campaign author to exactly determine the budget to be used in the campaign. The participants have equal chances to win the gift(s), however in the end only one or some lucky data collectors will actually receive a payout (i.e., the gift(s)).
- Mechanism $M_{2}$—Fixed micro-payment: A data collector is paid a fixed amount of money at the time he/she submits a valid answer of a campaign. This fixed amount of money is specified by the campaign author. In principle, the campaign author does not know the overall budget beforehand; yet this mechanism offers fair payments for the data collectors, i.e., they all get the same payment for each valid submission. To avoid an out of control overall cost, campaign authors may slightly restrict the fixed micro-payment scheme. For example, the campaign author can estimate the overall budget in advance, based on the number of data collectors and their historical behaviors [61], and calculate/adjust the amount of the micro-payment accordingly, or he can restrict the amount of micro-payouts, in correspondence with the desired amount of responses, until a predefined overall budget has been spent.
- Mechanism $M_{3}$—Variable micro-payment. The campaign author specifies the overall budget for the campaign; this budget is later divided by the number of valid submissions of the campaign, and each data collector gets an equal reward. While this mechanism allows the campaign author to know in advance the amount to spend, the data collectors do not know their payout beforehand, yet their reward is fair: they all get the same payment for each valid submission.

Using the campaign manager, the campaign author selects the incentive mechanism(s), which then computes and allocates the reward(s) to the participants of a particular campaign. Incentive mechanisms can be combined; a campaign may reward participants through multiple incentive mechanisms at the same time. For each mechanism, the campaign author specifies its necessary parameters through the campaign manager:

- Mechanism $M_{1}$: the number and the description of each of the (monetary) gifts being used in the lottery.
- Mechanism $M_{2}$: the micro amount to be paid for each valid submission.
- Mechanism $M_{3}$: the total budget for the campaign.

## 4. The Experiment

### 4.1. The Goals of the Experiment

The experiment aims to examine the behaviors of a large number of the data collectors who are simultaneously exposed to a variety of sensing campaigns and stimulated by the use of different types of monetary incentives. Specifically, through the Citizense framework, data collectors will face an expanding list of realistic sensing tasks that address issues relevant in the modern city context; to some of these campaigns, monetary incentives are applied. We seek to investigate the effectiveness of monetary incentives in general and identify the incentive mechanism(s) that is/are most successful in enhancing the participation of data collectors.

### 4.2. Setup and Progress of the Experiment

**Figure 3:**
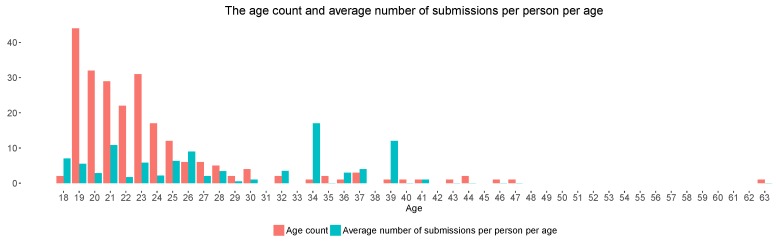
The age count and average number of submissions per person per age, for campaigns $C_{1}$–$C_{4}$.

The Citizense framework was deployed in the campus of Universitat Jaume I (Spain), as a surrogate of a smart city, targeting its students, professors, staff and visitors/passers-by. The experiment lasted 20 days, of which 14 were weekdays and 6 were in the weekend; it started with a promotional campaign aimed at recruiting participants: invitation emails were sent to the whole university community (academic and administrative staff, students), flyers were distributed and posters were placed in common, frequently-visited places inside the campus (e.g., bus stop, university restaurant & coffee shops, common meeting places, etc.). Participants could freely register, create campaigns of their choice and, participate in the campaigns created by the experiment organizer and other participants. Throughout the course of the experiment, there were 230 data collectors participating in the campaigns facilitated by the Citizense framework, of which 131 are females (56.9%) and 99 are males (43.1%). The data collectors’ ages range from 18 to 63, with a mean value of 23.47 and standard deviation of 11.72 (more details are provided in Figure 3). In other words, and confirmed by visual inspection, the majority of the data collectors are young adults (aged 18–25): students of the university; the rest includes older adults who are Ph.D students, lecturers, university staffs and passers-by. The educational background of the data collectors varies as they study at or are associated with different faculties of the university, namely technology and experimental sciences, law and economics, humanities and social sciences and health sciences. Most of the data collectors are locals; 7 data collectors are international students. All data collectors gave their informed consent for inclusion before they participated in the study, and the experiment complied with the ethics regulations of Universitat Jaume I (approval reference number 04/2018). Campaigns were launched regularly: among the 44 campaigns launched during the experiment, 34 campaigns originated from the participants who wished to create their own participatory sensing process and were created and launched during the course of the experiment. These campaigns addressed the participants’ concerns such as entertainment, food consumption, transportation and social issues; they differed from each other in content, requirements and complexity. The other 10 campaigns were created by the organizers of the experiment, and these campaigns addressed topics of general interest to the public. As a result, over the 44 campaigns, the data collectors had a wide range of campaigns to select from at any given time, the campaigns differed from each other in terms of incentives and content.

As studying user behavior under the presence of monetary incentives is the main focus of this work, 7 of the 10 campaigns originated from the organizers were selected to have associated incentives; we further refer to these campaigns as $C_{1}$, $C_{2}$, $C_{3}$, $C_{4}$, $C_{5}$, $C_{6}$ and $C_{7}$ (for an overview, see Table 1). They were available in both the local language (Spanish) and English, and offered a good spectrum of complexity and length: some campaigns contain a couple of atomic tasks without any conditional transitions while others present data collectors with a considerably longer list of tasks connected by conditional transitions. Campaigns $C_{1}$, $C_{2}$, $C_{3}$, $C_{4}$ were launched on day 1 of the 20 day experiment; $C_{5}$, $C_{6}$ on day 8; and $C_{7}$ on day 14. We intentionally selected this launching schedule to observe the behavior of the data collectors over time, which is later discussed in Section 5.1. Campaigns $C_{6}$ and $C_{7}$ were one time campaigns (i.e., a data collector can complete a one-time campaign only once); other campaigns could be completed multiple times. Table 1 gives an overview of the details of campaigns $C_{1}$–$C_{7}$. Each of these 7 campaigns was replicated into four functionally identical versions, where to each version one of the three aforementioned incentive mechanisms ($M_{1}$, lottery; $M_{2}$, fixed micro-payment and $M_{3}$, variable micro-payment), or the fourth mechanism $M_{4}$, a control mechanism which consists of no incentive (i.e., no reward), was applied. For each of these 7 campaigns, each data collector received only one version of an incentivized campaign (i.e., $M_{1}$–$M_{4}$), randomly distributed and permanently assigned. As such, the data collectors, for each campaign, were divided into four randomly distributed groups of equal size, where each group was assigned with a particular incentive mechanism. As a result, each individual data collector obtains a fixed set of incentivized campaigns throughout the experiment, where each incentivized campaign is associated with a random incentive mechanism. Based on post-random assignment observation, the authors confirmed that all data collectors were exposed to different incentive mechanisms, for different campaigns, and were thus aware of all the incentive mechanisms used in the experiment. For example, participant $P_{1}$ may receive the set of campaigns/incentive mechanisms ($C_{1}$ $M_{1}$, $C_{2}$ $M_{2}$, $C_{3}$ $M_{4}$, ...); participant $P_{2}$ may receive ($C_{1}$ $M_{4}$, $C_{2}$ $M_{1}$, $C_{3}$ $M_{2}$, ...); participant $P_{3}$ may receive ($C_{1}$ $M_{1}$, $C_{2}$ $M_{4}$, $C_{3}$ $M_{3}$, ...); etc. Participants $P_{1}$, $P_{2}$ and $P_{3}$ are all permanently assigned with their list of incentivized campaigns for the whole course of the experiment. We note that this experiment hereby differs from previous works in the fact that data collectors received different realistic sensing tasks with different incentive mechanisms at the same time.

During the course of the experiment, data collectors may view the list of currently available campaigns. In this list (see Figure 2 for a snapshot), campaigns with incentive mechanisms are marked with an icon to indicate the presence of an incentive (a Euro sign for a fixed or variable micro-payment, a gift icon for a monetary gift in the form of a voucher) and the exact incentive mechanism used (indicated by the number of the incentive between bracket); exact details of the incentive (i.e., the amount of payout) are at this point not yet visible. Campaigns without incentive do not feature an incentive logo. In order to prevent bias, the list of campaigns is shuffled every time the data collectors view their list of campaigns (i.e., refresh the campaign list). When the data collector selects a campaign, he/she will be shown the description of the campaign (see Section 3.4), and full details of the incentive mechanism. Specifically, for $M_{1}$, the data collectors are informed about the value of the monetary gift (i.e., in our experiment, for each campaign $C_{1}$–$C_{7}$ with $M_{1}$ assigned, a single monetary gift of 20 Euro was provided); prizes were raffled at the end of the campaign. For mechanism $M_{2}$, the data collectors are informed about the amount of the fixed micro-payment per valid submission which is paid upon submission (in our experiment, 25 Euro cents). For mechanism $M_{3}$, the data collectors are informed about the moment when their micro-payment is allocated (in our experiment, at the moment the time-limited campaign is finished); at this moment a fixed budget (in our experiment, 20 Euro) is equally distributed over valid submissions and participants are informed of their payout. After viewing the details of each campaign, with or without incentive mechanism, data collectors can choose whether or not to continue with the campaign in question or go back the list of campaigns and select another one.

During the experiment, the data collectors can instantly view their earned rewards (cash and monetary gifts): for example, they can see the increased amount of their earned cash right after completing a campaign with incentive mechanism $M_{2}$ or after the deadline of a time-limited campaign with incentive mechanism $M_{3}$. They are constantly informed that the rewards (i.e., cash and gifts) are deliverable immediately after the experiment. For every campaign in this experiment, we set up various metrics for further analysis, described in the next section: the number of times a campaign is viewed (in the campaign list), the number of times a campaign is opened and the number of times a campaign is submitted (by all data collectors).

## 5. Results

This section is devoted to the presentation and analysis of the outputs of the experiment in order to understand the data collectors’ behaviors under the presence of incentives, regardless of the campaign title, campaign content and the data collectors’ (general) desire. We hereby focus on campaigns $C_{1}$–$C_{7}$, which were specifically set up to study exactly this. We first present the raw data and a descriptive analysis (Section 5.1). Next, we perform a statistical analysis over the full set of participants, in order to determine the overall preferences for incentive mechanisms (Section 5.2). Subsequently, we analyze the behavior for specific relevant clusters, based on demographic parameters. We thus consider male versus female (Section 5.3) and younger (students) versus older (employees) (Section 5.4), to verify if any specific behavior occurs within these subsets of the experiment’s population.

### 5.1. Raw Data and Descriptive Analysis

In this section, the number of times that campaigns with incentives were viewed (in the campaign list), opened, and submitted are detailed. To visualize this raw data, we use stacked bar charts with bottom parts (dark color) indicating weekday list view/open/submissions and top parts (light color) showing weekends.

**Figure 4:**
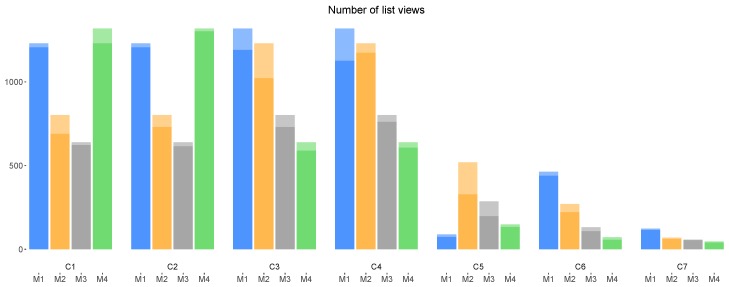
The number of list views of the campaigns. For each individual bar, dark (**bottom part**) and light color (**top part**) represent weekdays and weekends, respectively.

Figure 4 shows the number of list views from the four versions of a campaign (i.e., with incentive mechanisms $M_{1}$–$M_{4}$), for all campaigns $C_{1}$–$C_{7}$. Please note the differences in number of list views among the 4 versions of the same campaigns, for all campaigns. As mentioned before in Section 4.2, when a data collector views a (fixed) set of campaigns, the numbers of list views of all these campaigns will be incremented by 1. Therefore, the difference among the numbers of list views can be attributed to the more active participation of certain data collectors, who repeatedly view their assigned list of campaigns, thereby increasing the numbers of views of their assigned campaigns with associated incentive mechanism. The different durations among the campaigns result in the differences in number of overall views among $C_{1}$–$C_{4}$, $C_{5}$–$C_{6}$ and $C_{7}$; each campaign in the first group received many more views than that of the second group and third group, respectively.

**Figure 5:**
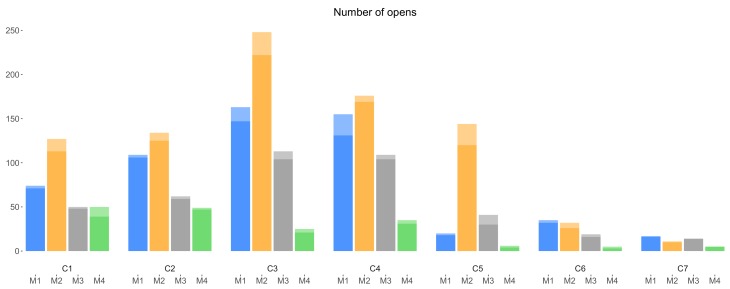
The number of opens of the campaigns. For each individual bar, dark (**bottom part**) and light color (**top part**) represent weekdays and weekends, respectively.

Figure 5 shows the number of opens from each version of a campaign, for all the campaigns $C_{1}$–$C_{7}$. From this figure, it is clear that in most cases, campaigns with mechanism $M_{2}$ were opened more than campaigns with other mechanisms. From Figure 4 and Figure 5, although campaigns $C_{1}$ and $C_{2}$ with mechanism $M_{4}$ were presented (viewed) more to the data collectors (more visible) than the same campaigns with other incentive mechanisms (i.e., $M_{1}$, $M_{2}$ and $M_{3}$), these campaigns ($C_{1}$ and $C_{2}$ with $M_{4}$) have been opened only the same amount of times as $C_{1}$ and $C_{2}$ with mechanism $M_{3}$, and fewer than with mechanism $M_{1}$ and $M_{2}$. In the same way, despite the fact that campaigns $C_{3}$ and $C_{4}$ with mechanism $M_{4}$ received slightly less number of list views than $C_{3}$ and $C_{4}$ with $M_{3}$, the former ($C_{3}$ and $C_{4}$ with $M_{4}$) received much less number of opens compared with the latter ($C_{3}$ and $C_{4}$ with $M_{3}$). This observation is a first indication that campaigns lacking an incentive ($M_{4}$) are less attractive to the data collectors. Finally, as for list views, we might observe an overall decrease in the total amount of opens among each campaign of the groups $C_{1}$–$C_{4}$, $C_{5}$–$C_{6}$ and campaign $C_{7}$, explainable by the subsequent shorter duration of campaigns.

**Figure 6:**
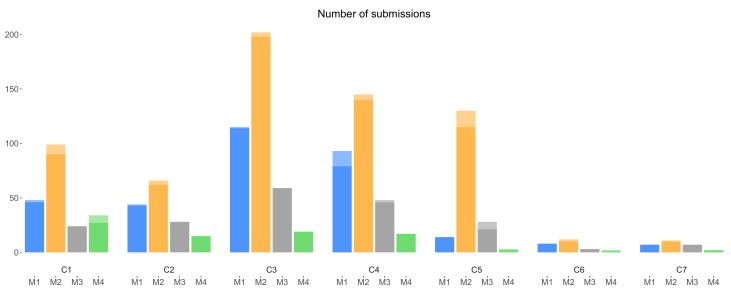
The number of submissions of the campaigns. For each individual bar, dark (**bottom part**) and light color (**top part**) represent weekdays and weekends, respectively.

Figure 6 shows the number of submissions from each version of a campaign with incentives, for all the considered campaigns. Similar to Figure 5, Figure 6 shows the higher performance of incentive mechanism $M_{2}$ compared with other mechanisms, which is analyzed later in Section 6.1. We can also again observe the lower number of submissions from $C_{5}$, $C_{6}$ and especially $C_{7}$ compared with that of $C_{1}$–$C_{4}$, which can be explained by their later launching dates and shorter durations.

Considering Figure 4, Figure 5 and Figure 6, we make two overall observations. First, we note that the amount of weekend interactions in all three charts is minimal, compared to weekday interactions; participants were thus notably less active during weekends, and weekend interactions thus do not appear to influence the overall (all days; weekday + weekend) trends. Second, there is an overall decrease in the number of interactions toward the end of the experiment; data collectors were not as active as in the beginning of the experiment. The decrease of participation in later campaigns can be partly explained by the diminishing duration of these campaigns ($C_{1}$–$C_{4}$ 20 days, $C_{5}$–$C_{6}$ 12 days and $C_{7}$ 6 days). However, extrapolating the duration may not fully explain the observed effect. This is also reflected when considering daily average participation. For example and based on daily average, the first 4 campaigns launched, two campaigns launched later and the last campaign receive 13.55, 9.20 and 5.16 submissions, respectively. Thus, there might be other factors that affect the data collectors’ interest in participating toward the end of the experiment. It can be considered that wearing out of novelty and lack of continuous promotion may have contributed to this downward trend in the number of submissions.

**Figure 7:**
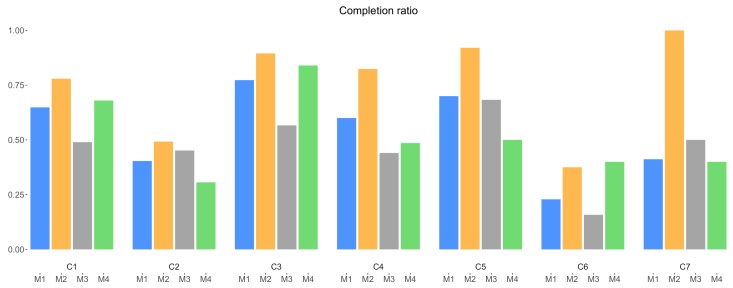
The completion ratios of the campaigns based on all days.

Based on the three values (list view, open, submission), additional descriptive ratios are calculated. These ratios are calculated using the overall number of list view, open and submission of the corresponding campaigns (all days, i.e., weekdays + weekend) (in Section 5.2, we show there is no significant difference in results when considering weekdays only versus all days). The completion ratio is defined as the ratio between the number of submissions and the number of opens of the same campaign. This ratio tells how an incentive mechanism potentially drives the data collectors to complete a particular campaign once they opened the campaign and learned the details of the incentive, and is depicted in Figure 7. In this figure, the completion ratio reaches 1 for mechanism $M_{2}$ in campaign $C_{7}$. It can be explained by the fact that only the most motivated data collectors were still actively using the application at the end of the experiment, and they took their last chance to immediately earn the corresponding reward. In general, mechanism $M_{2}$ achieved a higher completion ratio than the other three mechanisms. This can be explained by the fact that data collectors apparently prefer a fixed, certain reward, compared to an uncertain award. The situation for the other mechanisms is more mixed, with $M_{1}$ seemingly having a slight edge over $M_{3}$ and $M_{4}$ possibly due to the fact that data collectors prefer, in lack of a fixed micro payout, to have a chance to win a valuable gift, than waiting for an unknown amount or no award. Interestingly, and in contrast to the general completion and attraction ratio (discussed further), mechanism $M_{4}$ seems to perform well in some campaigns ($C_{1}$, $C_{3}$ and $C_{6}$): once opened, there is a high possibility the data collector will also complete the campaign. This can be explained by the fact that data collectors, despite the lack of incentive, might be attracted by the content of these campaigns, and therefore open and complete them.

**Figure 8:**
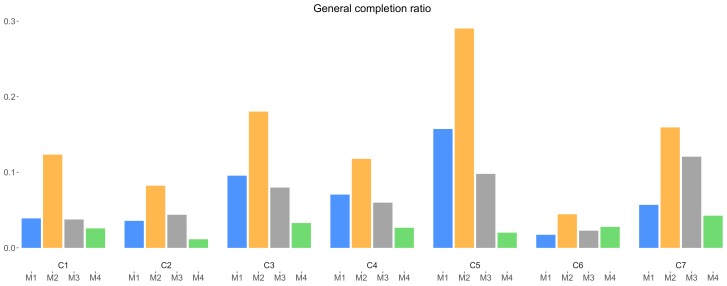
The general completion ratios of the campaigns based on all days.

The general completion ratio is the ratio between the number of submissions and the number of list views of the same campaign. This ratio shows how the presence of an incentive mechanism drives the data collectors to complete a particular campaign. Figure 8 depicts the values of the general completion ratio among campaigns. In this figure, $M_{4}$ generally is least effective, showing clearly how the presence of an incentive drives data collectors to contribute, and conversely, the lack of incentive deters them to contribute. On the other hand, $M_{2}$ maintains its top position, showing that fixed micro-payment seem to drive data collectors more to contribute than other mechanisms. As before, the situation for $M_{1}$ and $M_{3}$ is less clear. In general, data collectors seem to be more interested in completing a campaign which surely provides them with rewards immediately, and do not appreciate getting nothing.

**Figure 9:**
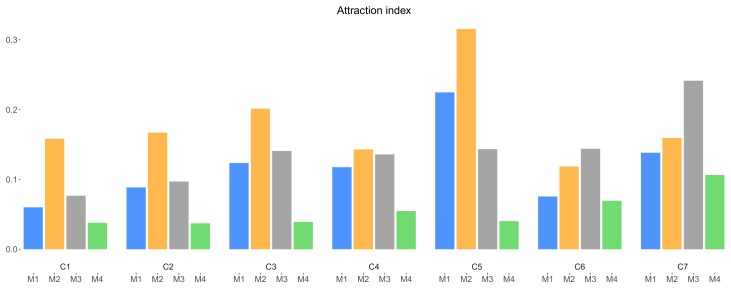
The attraction indexes of the campaigns based on all days.

The attraction index is the ratio between the number of open and the number of list views of the same campaign. It represents the effectiveness of an incentive mechanism in attracting data collectors to open a particular campaign (yet without knowing its exact incentive details), and conversely, their low interest in campaigns without incentive. Figure 9 depicts the values of the attraction indexes among the versions of the campaigns with incentives. While mechanism $M_{2}$ generally seems to outperform other mechanisms and $M_{4}$ has the least successful performance, mechanism $M_{3}$ generally slightly beats $M_{1}$ in attracting data collectors to click on a campaign when they see it in the campaign list. It seems data collectors are more curious how much money is distributed over data collectors, perhaps hoping for a certain and potentially relatively high payout, compared to knowing what the (value of the) gift is. However, as the completion ratio already showed (Figure 7), once opened, there is no clear advantage of $M_{3}$ over $M_{1}$ (rather on the contrary). This might suggest that, once realizing the potential gains (and perhaps being disappointed) of the variable incentive mechanism $M_{3}$, users have a slight preference to complete a campaign with a chance for high payout.

Finally, in campaign $C_{7}$, we performed a final questionnaire among data collectors to qualitatively assess their opinion regarding incentive mechanisms. First, on the question “Which type of incentives do you prefer?”, among the 31 respondents, 24 respondents selected “monetary rewards”, 6 respondents selected “no rewards needed” and 5 respondents selected “intangible rewards”. From the 24 respondents who selected “monetary rewards”, in a follow-up question, 15 indicated they preferred fixed micro-payment ($M_{2}$), 7 respondents indicated that any monetary incentive mechanism is fine, 1 respondent selected lottery-style payout ($M_{1}$) and 1 respondent selected variable micro-payment ($M_{3}$). Even though the questionnaire was performed on a relatively small sample (31) of the total amount of participants, it indeed qualitatively confirms that a majority of data collectors prefer a monetary incentive (compared to no or intangible incentives), and a majority prefers a fixed micro-payment as concrete monetary incentive mechanism.

### 5.2. Overall Statistical Analysis

This section is dedicated to the overall statistical analysis of the raw data presented in the previous section, in order to discover the general trends for the whole set of participants with respect to monetary incentive mechanisms. To this aim, we analyze the effectiveness of monetary incentive mechanisms by comparing different metrics of campaigns with incentives with those of the same campaign without incentive. We particularly focus on the number of submissions, as this is the final and most important step in the data collecting process. For this analysis, we consider two variants, only weekdays and all days (weekends + weekdays), in order to verify if the changing routines and patterns of user behavior during weekends influence the overall findings in participatory sensing behavior toward incentives. We first verify if there is a preference for certain incentive mechanism(s) when opening a campaign:

- Hypothesis H1: Data collectors do not have a preference when **opening** a campaign $C_{i}$ (with 1 ≤ *i* ≤ 7), with respect to the different incentives $M_{j}$ (with 1 ≤ *j* ≤ 4).

We hereby consider two variants of H1, namely considering all data (denoted as Hypothesis $H1_{all}$) and considering only weekdays (denoted as Hypothesis $H1_{weekdays}$). We will consistently use this notation for further hypothesis in the article.

In order to test if the data in question are indeed independent, we perform a $\chi^{2}$ test by considering significance level 0.05 on the number of campaign opens, per campaign $C_{i}$ and for each incentive mechanism $M_{j}$. For the full time period, the result of the $\chi^{2}$ test (*p*-value) for the campaigns with incentives ($C_{1}$–$C_{7}$) is less than 1 × 10^−6^ for $C_{1}$–$C_{6}$, and 0.082 for $C_{7}$; for weekdays only, the $\chi^{2}$ test gives similar results: *p*-values are less than 1 × 10^−5^ for $C_{1}$–$C_{6}$ and 0.09 for $C_{7}$. Consequently, we cannot accept Hypothesis $H1_{all}$ nor $H1_{weekdays}$ for all the campaigns (*p*-value < 0.05), except for $C_{7}$: there are significant differences among the number of opens of the four versions of each campaign $C_{1}$–$C_{6}$. In other words, data collectors do have a preference regarding the presence of an incentive when they open any of these campaigns, regardless of the campaign content. In contrast, there is no significant difference between the numbers of opens of the four versions of campaign $C_{7}$. It can be explained that $C_{7}$ was launched in the last week of the experiment when there were much less data collectors, as a result there is not enough data to show statistical significance. We now turned to test the following hypothesis:

- Hypothesis H2: Data collectors do not have a preference when **completing** a campaign $C_{i}$ (with 1 ≤ *i* ≤ 7), with respect to the different incentives $M_{j}$ (with 1 ≤ *j* ≤ 4).

Similarly and per campaign $C_{i}$, we perform a $\chi^{2}$ test by considering significance level 0.05 on the number of submissions of the four variants of the campaign ($M_{1}$–$M_{4}$) for the full 20-day period (verifying Hypothesis $H2_{all}$) and the weekdays (verifying Hypothesis $H2_{weekdays}$). For the former, the results of the $\chi^{2}$ test (*p*-value) is less than 1 × 10^−6^ for $C_{1}$–$C_{5}$, 0.016 for $C_{6}$ and 0.11 for $C_{7}$, and for the latter, the *p*-values are are less than 1 × 10^−6^ for $C_{1}$–$C_{5}$, 0.021 for $C_{6}$ and 0.16 for $C_{7}$. Consequently, we cannot accept Hypothesis $H2_{all}$ nor $H2_{weekdays}$ for all the campaigns (*p*-value < 0.05), except for $C_{7}$, and we conclude that there are significant differences among the number of submission of the four versions of each campaign $C_{1}$–$C_{6}$. In other words, participants do prioritize certain incentive mechanisms when they complete any of these campaigns with incentives, regardless of the campaign content. In contrast, there is again no significant difference between the number of submissions of the four versions of campaign with incentives $C_{7}$, mainly due to lower amount of data collectors participating at the end.

There is thus a statistically significant difference among different incentives, both in exploring (opening) and completing campaigns. Furthermore, the raw data (see Section 5.1) suggests that the presence of incentives seems to positively affect the behaviors of the data collectors (i.e., $M_{4}$ performs worst), and that among the incentive mechanisms, $M_{2}$ performs best. Consequently, there is a further need to compare the incentive mechanisms themselves, in order to determine which incentive outperforms another. For this comparison, we selected the campaigns $C_{1}$, $C_{2}$, $C_{3}$ and $C_{4}$ as these were exposed to all the data collectors for the same amount of time (i.e., throughout the full length of the experiment). We first confirm a statistical difference in opening campaigns with different incentive mechanisms, over all considered campaigns:

- Hypothesis H3: Data collectors **open** the campaigns $C_{i}$ (with 1 ≤ *i* ≤ 4) irrespective of the incentive mechanisms $M_{j}$ (with 1 ≤ *j* ≤ 4).

A non-parametric Kruskal-Wallis test (used for comparing two or more independent samples of equal or different sample sizes) on the number of opens of campaigns $C_{1}$–$C_{4}$ gives the result *p*-value of 0.009 for all days and 0.006 for weekdays. Therefore, Hypothesis $H3_{all}$ and $H3_{weekdays}$ cannot be accepted (*p*-value < 0.05) and the differences between the incentive mechanisms as perceived by the data collectors are confirmed. In other words, at least one mechanism statistically dominates another one. In a further step, we make a pair-wise comparisons of the incentive mechanisms based on the number of opens, in order to determine which incentive mechanism is statistically more attractive. For this purpose, the Mann-Whitney U test (used to determine whether two independent samples were selected from populations having the same distribution) was performed on each pair of incentive mechanisms, using the number of opens of campaigns $C_{1}$–$C_{4}$. Table 2 details the results of these statistical tests.

The results (*p*-value) in Table 2 again confirm the differences among the performances of the incentive mechanisms in attracting the data collectors to open campaigns, both during the whole experiment and the weekdays. The trends of the results are very similar for both of these two aforementioned time periods, with respect to the significance level of 0.05. Specifically, there are significant differences in performance between the pairs of incentive mechanisms $M_{1}$ and $M_{4}$, $M_{2}$ and $M_{4}$ and $M_{3}$ and $M_{4}$ and $M_{2}$ and $M_{3}$. We can conclude that mechanisms $M_{1}$, $M_{2}$, $M_{3}$ are all more effective than $M_{4}$; in other words, incentive mechanism $M_{4}$ has inferior performance and is thus least effective in driving the data collectors to open a campaign. Among mechanism $M_{1}$, $M_{2}$ and $M_{3}$, we can only conclude that $M_{2}$ is more effective than $M_{3}$; no statistical difference could be found between mechanism $M_{1}$ on one hand, and $M_{2}$ or $M_{3}$ on the other hand. Nevertheless, the latter, in combination with the dominance of $M_{2}$ over $M_{3}$, indicates that $M_{1}$ must necessarily lie in between $M_{2}$ and $M_{3}$. Indeed, this is confirmed by the overall average amount of openings for each incentive mechanism (see Table 3), and we can thus conclude a general descending order $M_{2}$ > $M_{1}$ > $M_{3}$ > $M_{4}$ in terms of their effectiveness in stimulating campaign openings, for the whole duration of the experiment and for the weekdays.

In the same way, we compare the effectiveness of the incentive mechanisms in driving the data collectors to complete and submit sensing campaigns. We first confirm a statistical difference in completing campaigns with different incentive mechanisms, over all considered campaigns:

- Hypothesis H4: Data collectors **complete** the campaigns $C_{i}$ (with 1 ≤ *i* ≤ 4) irrespective of the incentive mechanisms $M_{j}$ (with 1 ≤ *j* ≤ 4).

A non-parametric Kruskal-Wallis test on the number of submissions of campaigns $C_{1}$–$C_{4}$ gives the result *p*-value of 0.008 for the overall period and 0.009 for weekdays. Therefore, Hypothesis $H4_{all}$ and Hypothesis $H4_{weekdays}$ cannot be accepted (*p*-value < 0.05); these non-acceptances once again confirm the differences between the incentive mechanisms as perceived by the data collectors. The result of this test also indicates that at least one mechanism statistically overwhelms another one.

We then make pair-wise comparisons of the incentive mechanisms based on the number of submissions of these aforementioned campaigns to determine which incentive mechanism is more effective in pushing data collectors to complete campaigns. As before, the Mann-Whitney U test was performed on each pair of incentive mechanisms, using the number of submissions of campaigns $C_{1}$–$C_{4}$. Table 4 details the results of these statistical tests.

The results in Table 4 confirm the different performances of the incentives in pushing data collectors to complete campaigns. The trend of the results are identical for both the full time period and for weekdays, with respect to the significance level of 0.05. In particular, the pairs $M_{1}$ and $M_{4}$, $M_{2}$ and $M_{4}$ and $M_{3}$ and $M_{4}$, and $M_{2}$ and $M_{3}$ have significant differences, as suggested by the corresponding *p*-values. Interestingly, these are the same pairs that have significantly different performance for opening campaigns, as indicated in Table 2. Based on the p-values in Table 4, we can conclude that $M_{4}$ is least effective, and $M_{2}$ is more effective than $M_{3}$ in motivating data collectors to complete a campaign. The statistical test does not suggest any significant difference between $M_{1}$ on one hand, and $M_{2}$ and $M_{3}$ on the other hand. Nevertheless, the latter, in combination with the dominance of $M_{2}$ over $M_{3}$, indicates a general descending order $M_{2}$ > $M_{1}$ > $M_{3}$ > $M_{4}$ during the whole length of the experiment and the weekdays in terms of effectiveness of incentive mechanisms to entice users to complete campaigns. This order is confirmed when looking at the average amount of submissions per incentive mechanism, shown in Table 5.

As evidenced in this section, no different trends were found when including or excluding weekends from the data analysis. While Section 5.1 showed a much lower amount of activity during weekends, the different daily routines that participants exhibit during weekend (non-working) days does not influence their overall behavior toward incentive mechanisms in participatory sensing. Therefore, in the next sections, we only consider the full time period (20 days) of the experiment for further analysis.

### 5.3. Studying the Influence of Gender

After studying the overall tendency toward certain incentive mechanisms in the whole experiment’s population, and establishing a general order among incentive mechanisms, we now study the behavior of different demographic subgroups. In this section, we seek to analyze how the data collectors’ gender affects their behavior and the established general order among incentive mechanisms, using the number of submissions as the primary metric.

We first test for a statistically significant difference between the number of submissions of male and female data collectors:

- Hypothesis H5: Gender does not affect the number of submission when considering **completing** campaigns, for all campaigns and irrespective of incentive mechanisms.

As mentioned in Section 4.2, among the 230 data collectors there are 131 females and 99 males; they respectively submitted 1605 and 1180 times for all the 44 campaigns. Therefore, the average number of submission of a female data collector and a male data collector is 12.25 and 11.91, respectively. Concerning the campaign with incentives ($C_{1}$–$C_{7}$), female and male data collectors made 703 and 690 submissions, resulting in the individual average of 5.36 and 6.96 submissions, respectively. In order to determine statistically significant difference, the Mann-Whitney U test was used on the pair of individual female submission vector and individual male submission vector, which results in *p*-value 0.971, indicating very similar (close to 1) vector distributions. Consequently, the gender difference does not affect the general submission behavior of the data collectors.

Next, we verify Hypotheses H2 and H4, considering the full time period of the experiment, separately for male and female data collectors, in order to confirm/contradict the fact that not all incentive mechanism are considered equal (at least one mechanism dominates another), and the previously established general order among incentive mechanisms. For completeness and ease of comparison, we also repeat the results for all participants (male + female), as already provided in Section 5.2.

The $\chi^{2}$ tests on the number of submissions from female data collectors give the results (*p*-value) of less than 0.05 for campaigns $C_{1}$–$C_{6}$ and 0.21 for campaign $C_{7}$. Similarly, the same tests for the number of submission from male data collectors give the *p*-values of less than 0.05 for campaigns $C_{1}$–$C_{6}$ and 0.22 for campaign $C_{7}$. As a result, with the significance level of 0.05, we cannot accept Hypothesis $H2_{females}$ nor Hypothesis $H2_{males}$ for all the mentioned campaigns, except campaign $C_{7}$, again due to its short lifetime and low number of submission. Both female and male data collectors are thus stimulated by at least one incentive mechanism.

Considering the 4 campaigns $C_{1}$–$C_{4}$, to which all female and male data collectors where exposed throughout the full duration of the experiment, we first confirm a statistical difference in completing these campaigns with different incentive mechanisms by both female and male data collectors, for all considered campaigns. As in Section 5.2, the non-parametric Kruskal-Wallis tests on the combined number of submissions from female and male data collectors was used, resulting in *p*-values of 0.008 (female) and 0.003 (male), respectively. As a result, Hypothesis $H4_{females}$ and Hypothesis $H4_{males}$ cannot be accepted using the significance level of 0.05. These non-acceptances again confirm both the differences between the incentive mechanisms perceived by each gender-based group and the similar behaviors between these two genders.

For each gender, we then make pair-wise comparisons of the incentive mechanisms based on the number of submission of the aforementioned campaigns, using the Mann-Whitney U test. Table 6 details the results of these tests.

For female data collectors, the results of these tests are similar to those of all data collectors with respect to the significance level of 0.05. While the pairs $M_{1}$–$M_{2}$ and $M_{1}$–$M_{3}$ show no significant difference (*p*-value > 0.05), the other pairs do have significant difference between them. Following a similar reasoning as for the whole experiment population, and referring to Table 7 showing the average number of submission per mechanism from female data collectors, we can conclude an order of $M_{2}$ > $M_{1}$ > $M_{3}$ > $M_{4}$, which is the same order as found for the whole experiment population. However, for females, we observe a slightly more visible difference between $M_{1}$ and $M_{2}$ and a slightly smaller difference between $M_{1}$ and $M_{3}$, compared to those of the whole population; $M_{4}$ clearly lags behind.

For male data collectors, the tests show that only pairs $M_{1}$–$M_{2}$ and $M_{3}$–$M_{4}$ can be considered statistically equivalent; all other pairs show a significant difference (*p*-value < 0.05). This denotes that $M_{1}$ and $M_{2}$ are clustered on one end of the spectrum (performing better), and $M_{3}$–$M_{4}$ on the other hand of the spectrum (performing less effectively). In order to establish an order among these mechanisms in terms of their effectiveness, we referred to Table 7, where we notice the average number of submissions of $M_{2}$ is clearly larger than $M_{1}$, and $M_{3}$ larger than $M_{4}$, yet with a smaller absolute difference. The following order is thus supported: $M_{2}$ > $M_{1}$ > $M_{3}$ > $M_{4}$. This order is consistent with the order for all data collectors.

### 5.4. Studying the Influence of Data Collectors’ Age

A second demographic parameter, which is particularly relevant in the context of monetary incentives, is the age of participants. Particularly in a university campus, where a relevant amount of the population are students (which supposedly do not have an income), it might be worth studying if there is any difference between younger (students, no income) and older (possibly not students, with income) data collectors (note that, due to strict privacy regulations in Spain and ethical regulations in our university, we were not able to directly ask for income). Indeed, due to the relatively low monetary quantity of the used incentives, and due to participants’ different daily activities and schedule, their perception and behavior toward monetary incentives may be different depending on their age. Therefore, we consider younger participants (≤25), which generally are students, and older participants (>25) (consider that in Spain, students start university at 18, spend 4 years for a bachelor and 1 or 2 years for a master; possibly with an extra year. We verified the cut-off age by doing a similar analysis for cut-off age of 23 and 24, yielding the same results as 25). We hereby seek to analyze how the data collectors’ age (younger versus older), representing student (no income) and non-students (with income), affects their behavior and the established general order among incentive mechanisms, using the number of submissions as the primary metric.

First, we seek to determine if there is any correlation between age and amount of submissions:

- Hypothesis H6: There is no difference in the average number of submissions per person per age across the age spectrum, when **completing** a campaign, for campaigns $C_{1}$–$C_{4}$ and irrespective of incentive mechanism.

Figure 3 shows both the age of all data collectors and their average number of submissions per person per age from the four campaigns with incentives $C_{1}$–$C_{4}$. Visual inspection immediately reveals that this average number of submissions per person per age is quite variable, and decreases especially for older ages (not considering outliers of age 34 and 39). To statistically determine any correlation, we performed a Kendall rank correlation analysis, which measures the relationship of the average number of submissions per person per age and the data collectors’ age, in order to find the correlation between these two variables. This analysis considered the four campaigns with incentives $C_{1}$–$C_{4}$ which were exposed to all data collectors throughout the experiment. The Kendall correlation coefficient is −0.437 with the corresponding *p*-value of 0.002, meaning that there is a significant negative correlation between age and the average number of submissions per person per age. In other words, on average, older data collectors submit less results compared to their younger peers. The outliers in Figure 3 (age 34 and 39) all concern a single participant who submitted a large amount of answers for a single campaign, and thus do not represent a statistical interruption of the general trend.

In a further step, we aim to verify whether the previously established ordering for incentive mechanisms is valid for younger (≤25) and older (>25) data collectors. As for gender, we therefore verify Hypotheses H2 and H4, considering the full time period of the experiment, separately for younger and older data collectors. In the experiment, there were 195 younger data collectors and 35 older data collectors. Regarding all 44 campaigns available during the experiment, younger and older data collectors made 2446 and 339 submissions, resulting in the average number of submission of 12.54 and 9.68 per data collector, respectively. For the campaigns with incentives ($C_{1}$–$C_{7}$), younger and older data collectors submitted 1224 and 169 times, resulting in the individual average of 6.27 and 4.82, respectively.

The $\chi^{2}$ tests on the number of submissions from younger data collectors give the results (*p*-value) of less than 0.05 for all the 7 campaigns ($C_{1}$–$C_{7}$). Therefore, we cannot accept Hypothesis $H2_{younger}$ for all the considered campaigns; a preference for at least one incentive mechanism over another is present in all the considered campaigns. Note that when considering all data collectors (see Section 5.2—Hypothesis H2), we could not find a significant difference for campaign $C_{7}$. On the other hand, the $\chi^{2}$ tests on the number of submissions from older data collectors gives slightly different results: we found p-values of less than 0.05 for campaigns $C_{1}$–$C_{5}$, 0.11 for campaign $C_{6}$ and 0.57 for campaign $C_{7}$. Consequently, we cannot accept Hypothesis $H2_{older}$ for all the considered campaigns, except $C_{6}$ and $C_{7}$. In other words, older data collectors do have a preference regarding the presence of incentive for campaigns $C_{1}$–$C_{5}$, for the other two campaigns there is no significant difference among the four incentive mechanisms. Again, we note a slight variation: for all data collectors (see Section 5.2—Hypothesis H2), we did find a significant difference in incentive mechanisms for campaign $C_{6}$, while the insignificance for campaign $C_{7}$ for older people seems to compensate its significance for younger people. Similar as for earlier analyses, we note that the importance of these variations should not be overstated, as the shorter duration and lower participation of campaigns $C_{5}$–$C_{7}$ may play a role.

In the same way as previous analyses, more attention is paid on campaigns $C_{1}$–$C_{4}$ due to their longer exposure (20 days) to the data collectors and higher participation. We first confirm a statistical difference in the number of submissions of campaigns with different incentive mechanisms, for all campaigns, by younger and older data collectors. The Kruskal-Wallis test on the number of submissions from younger and older data collectors give the *p*-values of 0.012 and 0.034, respectively. As a result, Hypothesis $H4_{younger}$ and $H4_{older}$ cannot be accepted considering the significance level of 0.05. These non-acceptances again confirm the differences between the incentive mechanisms perceived by each age-based group, which need to be analyzed further. To do so, we then make pair-wise comparisons of the four incentive mechanisms based on the number of submissions of campaigns $C_{1}$–$C_{4}$, using the Mann-Whitney U test. Table 8 details the results of these tests, along with the results for all users, repeated from Section 5.2.

For younger data collectors, the results of these tests are similar to those of the whole set of data collectors with respect to the significance level of 0.05. The tests show that all pairs of incentive mechanisms have a significant difference, except the pairs $M_{1}$–$M_{2}$ and $M_{1}$–$M_{3}$. Table 9 conforms to these differences; the dominance of $M_{2}$ over $M_{3}$ suggests that $M_{1}$ lies between these two mechanisms, and all these three mechanisms all dominate $M_{4}$. Therefore, the general order among the incentive mechanisms can be described as $M_{2}$ > $M_{1}$ > $M_{3}$ > $M_{4}$.

For older data collectors, we observe a significant difference between $M_{4}$ and all other incentive mechanisms (*p*-value < 0.05), confirming that $M_{4}$ performs least effectively. However, we can no longer see any statistically significant preference among mechanisms $M_{1}$, $M_{2}$ and $M_{3}$: among incentives mechanisms, older data collectors do not seem to have a clear preference. Nevertheless, by referring to Table 9, we can establish, the general order among the incentive mechanisms as $M_{2}$ > $M_{1}$ > $M_{3}$ > $M_{4}$, although the gaps between $M_{2}$, $M_{1}$ and $M_{3}$ are not significant; $M_{4}$ is still significantly least desirable than any other mechanism. Again, we need to call for caution, as few older data collectors (smaller sample size)) were available; therefore, these results should be confirmed by repeated experiments.

## 6. Discussion

### 6.1. Further Analysis and Summary of Results

First of all, it is empirically clear that monetary incentives enhance the participation of the data collectors in the Citizense platform, both in terms of exploring (opening) campaigns and completing (submitting) campaigns. While the action of opening a campaign shows that a data collector is interested in that specific campaign, the submission matters more as that data collector finishes the sensing process and produces a result needed by the campaign author. To show the importance of incentives, it was first statistically confirmed using two different tests, i.e., by not accepting Hypothesis H1 and H2 (for campaigns $C_{1}$–$C_{6}$), and Hypothesis H3 and H4 (for campaigns $C_{1}$–$C_{4}$), that both when considering the campaigns separately, and when considering the campaign jointly, there is a statistically significant difference between the different incentive mechanisms, both in opening and completing campaigns. We furthermore confirmed that including or excluding weekend, in which data collectors exhibit a different schedule with different daily activities, does not influence the result of the analysis. In absolute terms, and in many cases, the numbers of opens and submissions of the campaigns that are applied with mechanism $M_{1}$, $M_{2}$ and $M_{3}$ (with incentive) are higher than those of the same campaign with mechanism $M_{4}$ (without incentive), regardless of the campaign content. This suggests that participants actively seek out and prefer campaigns with incentives, and is in line with one of the conclusions from [34]. We note that regarding quality (e.g., the ratio of blank submissions, the length of textual answers), we proportionally did not observe a difference between campaigns with or without incentive. This is an indication of the fact that data collectors generally did not abuse the framework to get extra rewards by submitting blank answers or low quality information, which is in line with conclusions from [62], where volunteers were doing similar tasks under the presence of material incentives.

Once statistically shown that there are significant differences in the performance of incentive mechanisms, we shifted our focus to establishing a ranking between different mechanisms: which monetary incentive mechanism performs better than another. Our analysis (the Mann-Whitney U tests using the number of opens and submissions) confirm what the absolute numbers already suggested: in all cases, campaigns with incentive mechanisms perform significantly better than campaigns without, both when exploring (opening) and completing (submitting) them. Among the monetary incentive mechanisms ($M_{1}$, $M_{2}$ and $M_{3}$), in absolute numbers (see Figure 5 and Figure 6), a fixed micro-payment (mechanism $M_{2}$) works more effectively than other incentive mechanisms, both to attract participants (opening campaigns) and to entice them to complete campaigns. Next in line is a lottery-based award, performing better than a variable micro-payment, yet we note that the completion ratio, and especially the general completion and attraction ratios show a mixed image; the latter even suggests a slight advantage for a variable micro-payment. This might indicate that even if initially attracted and opening a micro-payment campaign, participants do not appreciate the potentially low payout and do not complete the campaign. The statistical analysis (the Mann-Whitney U tests) however could only confirm the superiority of fixed micro-payment over variable micro-payment; no statistically significant difference could be shown between lottery-style incentive and both fixed and variable micro-payments. Nevertheless, by reasoning over the aforementioned statistically significant differences, and by comparing the overall average of openings/submissions per incentive mechanism, an overall descending ranking of $M_{2}$ > $M_{1}$ > $M_{3}$ > $M_{4}$ could be established.

The different ratios (completion ratio, general completion ratio and attraction index) paint a similar picture of the performance of the incentive mechanisms. These ratios shows how the data collectors are driven to participate in a campaign at different stages in their data collection process: exploring the list of campaigns and selecting a campaign, learning the full details of the campaigns and its associated incentive, and completing the campaign to be eligible for the reward(s). Among the incentive mechanisms, mechanism $M_{2}$ outperforms all other mechanisms in attracting data collectors to explore a campaign as well as complete it, regardless of the campaign content. Its top performance is clearly shown in Figure 7, Figure 8 and Figure 9. On the other side of the ranking, mechanism $M_{4}$ has the least successful performance; this outcome can be expected due to its no-reward mechanism. In between these two ends are the mechanism $M_{1}$ and $M_{3}$, they have comparable performances and there is no clear winner among these two mechanisms. It seems that mechanism $M_{3}$ (and obviously $M_{2}$) is a bit more effective in attracting data collectors to open a campaign than $M_{1}$, which is evident by the larger attraction index (Figure 9). However, mechanism $M_{1}$ slightly beats $M_{2}$ in driving data collectors to complete a campaign although the differences are usually not large.

**Figure 10:**
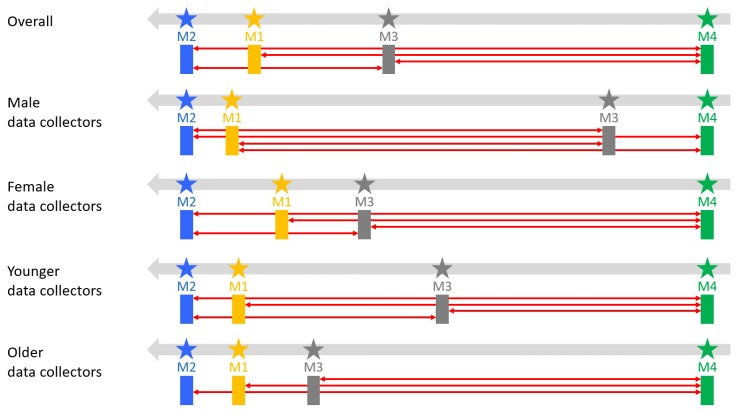
The approximate order of the incentive mechanisms, for the overall data collectors and their subgroups based on number of submissions. The significant differences (based on the *p*-values less than 0.05) between the mechanisms are marked by red arrows.

Once the general order of the incentive mechanisms was established for the complete set of data collectors, we shifted our focus to specific subgroups, based on demographic parameters, of the overall population: male versus female and younger versus older. Using the number of submissions, each subgroup’s behaviors of completing campaigns are first compared and then examined separately. Concerning the gender of the data collectors, it does not affect the behavior of a data collector completing campaigns. For both female and male data collectors, a general order of $M_{2}$ > $M_{1}$ > $M_{3}$ > $M_{4}$ can be established, although we observe difference between the different incentive mechanisms when ranking. In the mechanism ordering for male data collectors, $M_{2}$ and $M_{1}$ are at the top of the order with a small difference between each other, while $M_{3}$ and $M_{4}$ are at the bottom of the order with a significantly large difference from the two top ranks. For female data collectors, the three incentive mechanisms $M_{1}$, $M_{2}$ and $M_{3}$ are closer together, with $M_{4}$ performing significantly least effectively. Considering age, and using a cut-off age of 25, we could find the differences in the younger and older data collectors’ behavior of completing a campaign. Firstly, younger data collectors significantly submit more results than older peers, and we found a negative correlation between average number of submissions and age. Secondly, although younger and older data collectors share the same general order of the mechanism ($M_{2}$ > $M_{1}$ > $M_{3}$ > $M_{4}$), younger data collectors prefer most mechanism $M_{2}$, then $M_{1}$. Mechanism $M_{3}$ ranks further below the first two mechanisms, and at the bottom of the order is $M_{4}$ with a large difference to all other mechanisms. In contrast, the older data collectors are in favor of any kind of incentive mechanisms, hence the differences are not significant among $M_{1}$, $M_{2}$ and $M_{3}$; however $M_{4}$ is clearly at the bottom of the order with a significant difference compared to the other three mechanisms. We hereby note that the results for older data collectors should be interpreted with caution, as the total amount of older data collectors was relatively small; repeated experiments should confirm these findings. Figure 10 provides an overview of the ranking of incentive mechanisms, and their relative differences, for the whole population, and different considered subgroups.

In general, data collectors seem to choose the incentive mechanism that gives them concrete, tangible and predictable rewards rather than the incentive mechanism that has some uncertainties (fixed versus variable micro-payment), the possibility of a high payout (lottery) does seem to be appealing as well. This empirical finding is consistent with a qualitative evaluation performed through campaign $C_{7}$, which had a question that directly asked data collectors which incentive mechanism they prefer after they have been exposed to all the different mechanisms. The majority of respondents (62.5%) chose fixed micro-payment (mechanism $M_{2}$), 29.16% said that any reward is fine, 4.16% chose variable micro-payment (mechanism $M_{3}$) and 4.16% chose lottery-style mechanism (mechanism $M_{1}$). Nevertheless, we need to note that only 13.5% of the total amount of participants completed $C_{7}$, and thus the results should be interpreted with caution. More generally, the participation rate decreased toward the end of the experiment, as evidenced by the reduced participation to the campaigns launched toward the end of the experiment ($C_{5}$, $C_{6}$ and $C_{7}$). The prolonged participation of data collectors is indeed a challenge for participatory sensing campaign organizers, which should be mitigated (e.g., by continuous promotional campaigns). It is hereby interesting to mention that we observed a slightly increased interest in campaigns (opening and completing) immediately after a mobile phone notification was sent regarding the availability of new campaigns (two times throughout the experiment).

### 6.2. Advice for Holding Successful Participatory Sensing Campaigns

From the experience gained running our experiment, and the analyses performed on the result, we summarize the following advice to improve the success of participatory sensing campaigns:

- Promotion: initial promotion is key to gather a crowd willing to contribute, distribute the participatory sensing app and bring various campaigns under the public attention. However, continuous promotion over a longer period of time is as important, as participants gradually lose interest and there is a risk of them dropping out. Different strategies may be used for initial (i.e., traditional advertising) and continuous promotion (e.g., mobile phone notifications, recommended campaigns, incentives, …).
- Incentives work: participants like to be compensated for their efforts. Any kind of (monetary) incentive, even if very small or if there is only a chance on a reward, helps to increase response rate.
- Tangible rewards: Participants generally prefer tangible rewards, even if smaller, compared to an uncertain award. We recommend fixed micro-payments for qualitative submissions, possibly bound somehow to keep the campaign organizer’s budget foreseeable (e.g., limit rewards to the necessary minimum amount of required submissions), even though a lottery-style incentive may be an alternative.

### 6.3. Limitations

First, our case study might have suffered from the homogeneity of the participants. They are mostly university students, and to a lesser extent staff; they all have at least the education level of undergraduate studies. If the scope of the case study were bigger, i.e., extended to the whole city, a more demographically diverse background of participants can be achieved. Second, a larger amount younger participants (students) was observed compared to a much smaller number of older participants. While part of our study did consider younger and older participants separately, caution needs to be taken when interpreting the results of the older participants due to the smaller sample size. Ideally, repeated experiments for older participants should confirm the obtained results. Third, the number of campaigns with incentives was limited (7), and campaigns launched late in the experiment attracted few participants (3). A larger number of campaign with incentives would have given the data collectors more chances to interact with the campaigns and its incentive mechanisms, yielding the conclusions based on a larger dataset.

## 7. Conclusions

This article studied how the behavior of participants of a participatory sensing framework is influenced by the use of different monetary incentive mechanisms. In particular, through our Citizense participatory sensing framework, a variety of realistic participatory sensing tasks were deployed, using three monetary incentives: fixed micro-payment, variable micro-payment, and lottery style payout. In addition, a fourth control mechanism, no incentive/payout, was used. Fairness is a key characteristic in the incentive mechanisms: the mechanisms depend on criteria that are theoretically fair to all participants. These mechanisms were thoroughly tested in a real-world environment (a university campus), over a 20-day time period and with 230 data collectors.

By looking at the overall results, we see that the incentive mechanisms significantly increase the participation of the data collectors, while the framework maintains the quality of the submitted results: data collectors opened campaigns significantly more times and submitted more results when rewards through an incentive were available. Among the three implemented mechanisms, the statistical analysis shows that fixed micro-payment incentive (mechanism $M_{2}$) outperforms variable micro-payment. A qualitative evaluation, even though performed on a small portion of participants, confirmed that the majority of data collectors prefers a fixed micro-payment, over any other incentives. However, even though no statistically significant difference between the lottery-style payment latter and fixed/variable micro-payment could be found, the overall statistical analysis suggests an global decreasing performance ranking as follows: fixed micro-payment > lottery-style payout > variable micro-payment > no incentive. No difference in results was found when including or excluding weekends, showing that changed daily schedule and activities do not influence the overall results.

When considering different subgroups, according to demographical parameters (gender and age), the same performance ranking among incentive mechanisms could be found. However, different internal distances between individual incentive mechanisms have been observed. Most remarkably, male data collectors seem to only slightly prefer variable micro-payment over no incentive (no significant difference), and for older data collectors (>25), no significant difference could be found between any of the three incentive mechanisms, while they are all still significantly preferred to no-incentive mechanism. The latter however needs to be confirmed in repeated experiments, due to the smaller sample size of older data collectors. Generally, we also observed that there is a negative correlation between age and average amount of submissions: the older the participants, the less they submit on average.

Finally, based on the experience running our experiment, and the results obtained, we formulate three advices for participatory sensing campaign organizers: (1) continuous promotion is essential to attract participants and keep them interested; (2) monetary incentive mechanisms work better than not using an incentive, independent of type of incentive (3) we recommend predictable, tangible rewards (fixed micro-payment), rather than variable unpredictable rewards; a lottery-style reward may be an alternative.

This research performed a detailed, large-scale empirical study into the behavior of data collectors in the presence of monetary incentive mechanisms in participatory sensing. While this study investigated the overall trends, and studied particular subgroups based on gender and age, we call for further empirical research focusing on other/additional relevant factors. In particular, in our study, we focused on one particular subjective reason to participate (i.e., monetary incentive), and minimized the influence of other (subjective and objective) factors. As such, this research leaves ample opportunity for future research, building upon and further refining the conclusions drawn from our large-scale empirical study into incentive mechanisms. In particular, the influence of demographic factors (e.g., age, level of income, cultural aspects, etc.), and the interplay between objective (e.g., costs to participate) and subjective reasons (e.g., subject of campaign, purpose, privacy, etc.) needs to be further studied.

## Figures and Tables

**Table 1:** The different parameters of the campaigns with incentives.

Campaign	Theme	Duration	Remark
$C_{1}$	Graffiti in the city	20 days	
$C_{2}$	City incidents	20 days	
$C_{3}$	Different aspects of citizen participation	20 days	
$C_{4}$	WiFi signal strength	20 days	
$C_{5}$	Street animals	12 days	
$C_{6}$	Use of bicycle	12 days	One-time campaign
$C_{7}$	Final survey	6 days	One-time campaign

**Table 2:** The results of the Mann-Whitney U test for all pairs of incentive mechanisms, using the number of opens during the full period (all) and only weekdays.

Pairs of Mechanism	$M_{1}$–$M_{2}$	$M_{1}$–$M_{3}$	$M_{1}$–$M_{4}$	$M_{2}$–$M_{3}$	$M_{2}$–$M_{4}$	$M_{3}$–$M_{4}$
*p*-value (all)	0.34	0.2	0.029	0.029	0.029	0.029
*p*-value (weekdays)	0.2	0.2	0.029	0.029	0.029	0.029

**Table 3:** Average openings per incentive mechanism, for all campaigns $C_{1}$–$C_{4}$.

Incentive Mechanism	$M_{1}$	$M_{2}$	$M_{3}$	$M_{4}$
Average amount of openings (all)	125.25	171.25	83.5	39.75
Average amount of openings (weekdays)	113.75	157.25	78.75	34.5

**Table 4:** The results of the Mann-Whitney U test for all pairs of incentive mechanisms, using the number of submissions during the full period (all) and the weekdays.

Pairs of Mechanism	$M_{1}$–$M_{2}$	$M_{1}$–$M_{3}$	$M_{1}$–$M_{4}$	$M_{2}$–$M_{3}$	$M_{2}$–$M_{4}$	$M_{3}$–$M_{4}$
*p*-value (all)	0.2	0.2	0.029	0.029	0.029	0.029
*p*-value (weekdays)	0.2	0.2	0.029	0.029	0.029	0.029

**Table 5:** Average submissions per incentive mechanism, for all campaigns $C_{1}$–$C_{4}$.

Incentive Mechanism	$M_{1}$	$M_{2}$	$M_{3}$	$M_{4}$
Average amount of submissions (all)	77.75	133	41	19.25
Average amount of submissions (weekdays)	70.5	122.5	39.25	18.75

**Table 6:** The results of the Mann-Whitney U test for all pairs of incentive mechanisms, using the number of submissions from female and male data collectors.

Pairs of Mechanism	$M_{1}$–$M_{2}$	$M_{1}$–$M_{3}$	$M_{1}$–$M_{4}$	$M_{2}$–$M_{3}$	$M_{2}$–$M_{4}$	$M_{3}$–$M_{4}$
*p*-value (female)	0.14	0.24	0.021	0.021	0.021	0.021
*p*-value (male)	0.48	0.043	0.042	0.043	0.042	0.55
*p*-value (all)	0.2	0.2	0.029	0.029	0.029	0.029

**Table 7:** Average submissions per incentive mechanism by all female and male data collectors, for all campaigns $C_{1}$–$C_{4}$.

Incentive Mechanism	$M_{1}$	$M_{2}$	$M_{3}$	$M_{4}$
Average amount of submissions (female)	43	62.75	28	11.25
Average amount of submissions (male)	34.75	70.25	13	8
Average amount of submissions (all)	77.75	133	41	19.25

**Table 8:** The results of the Mann-Whitney U test for all pairs of incentive mechanisms, using the number of submissions from younger and older data collectors.

Pairs of Mechanism	$M_{1}$–$M_{2}$	$M_{1}$–$M_{3}$	$M_{1}$–$M_{4}$	$M_{2}$–$M_{3}$	$M_{2}$–$M_{4}$	$M_{3}$–$M_{4}$
*p*-value (younger)	0.343	0.2	0.029	0.029	0.029	0.029
*p*-value (older)	0.343	0.486	0.029	0.2	0.029	0.029
*p*-value (all)	0.2	0.2	0.029	0.029	0.029	0.029

**Table 9:** Average submissions per incentive mechanism by younger and older data collectors, for all campaigns $C_{1}$–$C_{4}$.

Incentive Mechanism	$M_{1}$	$M_{2}$	$M_{3}$	$M_{4}$
Average amount of submissions (younger)	69	116.25	34	16.75
Average amount of submissions (older)	8.75	16.75	7	2.5
Average amount of submissions (all)	77.75	133	41	19.25
